# Biochemical Impedance on Intracellular Functions of Vitamin B12 in Chronic Toxigenic Mold Exposures

**DOI:** 10.1100/tsw.2007.113

**Published:** 2007-10-12

**Authors:** Ebere C. Anyanwu, Ijeoma Kanu

**Affiliations:** ^1^ St. Peter's MCH, 190 College Road, Birmingham, UK; ^2^ Department of Microbiology, Abia State University, Nigeria

**Keywords:** Vitamin B12, toxigenic molds, mycotoxins, One-carbon metabolism

## Abstract

A majority of patients with neurological disorders with chronic exposures to toxigenic molds and mycotoxins has vitamin B12 deficiency that is unrelated to dietary insufficiency. Vitamin B12 is a source of coenzymes, and participates in intracellular recycling of methionine, and in methionine synthase reactions. The biochemical processes that lead to B12 depletion and deficiency are not fully understood. This paper examines and assesses various most likely biochemical reasons that could impede upon the normal intracellular functions of vitamin B12 that lead to neurological manifestations. By biochemical implications and derivations, it is most likely that mycotoxins interrupt the structure and function of vitamin B12 through reactive interference with the normal One-Carbon metabolism leading to the observed clinical neurological manifestations such as nerve damage and, demyelination, degeneration of PNS leading to paralysis, progressive peripheral neuropathy, and spinal degeneration.

## INTRODUCTION

Vitamins are among the major factors that affect human health and development, are the most essential. Vitamins are natural organic compounds that can be extracted from animal or vegetable sources or be chemically synthesized in the laboratory but required in trace amounts for health, growth, and reproduction[1]. They are in essence, often functionally coenzymatic and relatively more reactive than other macronutrients such as proteins, lipids, and carbohydrates. Although deficiency of a single vitamin is relatively uncommon, it can occur because of inborn error of metabolism, unusual restriction in dietary intake, or failure to absorb a single vitamin[2]. However, in the USA it was observed that a majority of patients presenting with chronic exposures to toxigenic molds had persistent vitamin B12 deficiencies, which were not related to dietary insufficiency[38]. These patients eat well and take appropriate nutrient supplements, however some of their main symptomatic and neurological manifestations are similar to those found in a typical vitamin B12 deficiency (Table 1a and 1b).

The reasons for the persistence of vitamin B12 deficiency is not understood however, it is proposed that the mycotoxins from the chronic toxic mold exposures must have interfered with major metabolic processes that regulate the biosynthesis, re-uptake and utilization of vitamin B12. The most common of these is the lack of intrinsic factor secretion by the stomach and intestinal malabsorption. Malabsorption may be caused by inflammatory diseases of the small intestine, intestinal stasis with overgrowth of colonies of bacteria[3] or toxigenic molds. More frequent are complex deficiencies that arise as a result of food fads; as complications of certain diseases, especially those that affect food absorption; as a result of massive losses of blood or from hemodialysis; and with use of certain drugs[1,3].

**Table 1a**
***Signs and symptoms of B12 deficiency***

| | |
|---|---|
| i) Headache, fatigue, loss of appetite | iv) Psychosis, confusion |
| ii) Pinky-red sore or smooth tongue | v) Depression, memory loss |
| iii) Growth failure in children | vi) Anxiety, insomnia |
| | ix) Trigeminal neuralgia, osteoarthritis |

**Table 1b**
**Clinical neurological Indications**

| | |
|---|---|
| i) Nerve damage and demyelination | v) Alzheimer’s disease, allergies, asthma |
| ii) Degeneration of PNS leading to paralysis | vi) Bipolar, chronic fatigue syndrome |
| iii) Progressive peripheral neuropathy | vii) Crohn’s disease Multiple sclerosis |
| iv) Spinal degeneration and macrocytic cells. | viii) Insomnia, Sciatic neuritis. |

A majority of patients with neurological disorders as a result of chronic exposures to toxigenic molds and mycotoxins has vitamin B12 deficiency that is unrelated to dietary insufficiency. The aim of this paper therefore, is to examine and assess various biochemical reasons that might have impeded upon the normal intracellular functions of vitamin B12 that lead to neurological manifestations.

## METHODS

Vitamins in general are first defined and the nutritional sources and functions of vitamin B12 are cited to enable a better understanding of how its interactions with one-carbon metabolism and other biological macromolecules, and how mycotoxins could interfere with these interactions. Available literatures on vitamin B12 deficiencies in chronic toxigenic mold exposures are searched and the possible synergistic relationships with mycotoxins in one-carbon metabolism are evaluated.

The processes that could lead to the manifestations of biochemical and neuropathological symptomology are reviewed. An outline of the mechanism by which mycotoxins could disrupt or interfere with the normal functions of vitamin B12 in one-carbon metabolism is proposed. The overall functions of vitamin B12 as a source of coenzymes, intracellular recycling of methionine, methionine synthase reaction, the prevention of chromosome breakage, methylation and in maintaining a one-carbon metabolic balance are reviewed.

## DEFINITION

Vitamin B12 (cobalamin) is a water-soluble vitamin that is stored in the liver and although sensitive to ultraviolet light vitamin B12 is derived from animal products including liver, beef, kidney, chicken, fish such as salmon, halibut and tuna, yogurt, milk, Swiss cheese, American processed cheese, and egg.

Vegetarians may derive vitamin B12 from cereals, Soya milk and vitamin B12 fortified vegetarian burger patties. In humans, the daily vitamin B12 requirement is approximately 0.5 micrograms. However, the recommended daily intake for adults is 2.0 micrograms daily for adults, about 2.2, and 2.6 micrograms during pregnancy and during breast-feeding respectively[3]. Vitamin B12 is essential for carbohydrate, fat and protein metabolism and serves an important role in the formation and regeneration of red blood cells, and in the maintenance of the central nervous system.

## The Role Mycotoxins in Vitamin B12 Deficiency

The problem of regulating mycotoxin biosynthesis, determining the factors that influence the extent of mycotoxin contamination of food and devising new methods of reducing or eliminating their contamination of food has become an important aspect of clinical and environmental mycology. Fungal metabolism lead to the production of secondary metabolites the majority of which are formed from a relatively small number of branch points of primary metabolic pathways. Invariably, some of these secondary metabolites are used as antibiotics while others are very toxic and carcinogenic to humans and animals. There are well over one hundred known mycotoxins many of them contaminate cereals, grains and many other foodstuffs worldwide. Additionally, several genera of fungi produce toxins in pasture grasses, producing a variety of problems directly in animals grazing in these pastures and indirectly in the farmers.

There are several pieces of evidence to show that mycotoxins affect cellular activities of the brain to which vitamin B12 plays a major role. For example, Riley et al.[4] showed that alteration of sphingolipid metabolism was associated with fumonisin-induced animal diseases including increased apoptotic and oncotic necrosis, and carcinogenesis in animal liver and kidney. It is believed that the biochemical consequences of fumonisin disruption of sphingolipid metabolism most likely to alter cell regulation are increased free sphingoid bases and their 1-phosphates, alterations in complex sphingolipids, and decreased ceramide (CER) biosynthesis.

Earlier, Enongene et al.[5] demonstrated the disruption of sphingolipid metabolism in small intestines, liver and kidney of mice dosed subcutaneously with fumonisin B(1). Vitamin B12 possibly interacts with mycotoxins to effects some of the biochemical and neurological changes such as was discussed by Crews et al.[6] It is not yet clear whether vitamin B12 deficiency precludes fungal infection or vice versa. However, the factors commonly believed to predispose to recurrent chronic atrophic fungal infections included deficiency in whole blood folate, iron and vitamin B complex[7]. These atrophies could occur because of metabolic disorders such as inactivity of enzymes that are characteristic of vitamin B complex deficiencies[8]. Vitamin B12 is implicated in the pathogenesis of several fungal infections such as candidaisis even though much disagreement exists as to the specific roles played by individual vitamins[9]. Nonetheless, there is little doubt that metabolic and nutritional factors either acting locally or via systemic mechanisms could significantly affect the pathogenesis of fungal infection[10].

## Mycotoxins, One-Carbon and Vitamin B12 Interactions

One-carbon metabolism is a complex set of biochemical reactions that contribute to many central biosynthetic pathways. Although, the regulation of this complex set of reactions is difficult to analyze because of the ability of the reactants to interchangeably supplement the pools of one-carbon-derived compounds, several approaches are adopted to identify the components of the signaling system that regulates the metabolic activity[11]. One-carbon metabolism is an essential process that relies on one-carbon donor molecules such as serine, glycine, and formate.

Cells regulate the balance of one-carbon flow between the donors by regulating cytoplasmic hydroxymethyltransferase activity of the donor in a side reaction. The knowledge of one carbon metabolism is essentially very important because any failure in the metabolic process is a risk factor for cardiovascular disease, stroke and thrombosis, loss of neurocognitive function and Alzheimer's disease, similar neurological and psychiatric disturbances including depression, dementia, and a demyelinating myelopathy[12,13,14].

To prevent the occurrence of these metabolic disorders, there has to be a mechanism for the one carbon metabolism to be homeostatically maintained and regulated. The effect of mycotoxins on one-carbon metabolism that lead to Vitamin B12 depletion or deficiency and the mechanism by which this happens, is not clear. It is envisaged that mycotoxins directly and indirectly may interfere with a number of vitamin B12 functions and one-carbon metabolism. By implication as shown in the outline (Figure 1) for example, improper function of the one-carbon metabolism can lead to abnormal fatty livers and increased liver-cell turnover thereby, promoting carcinogenesis as has been found in experimental animals[15].

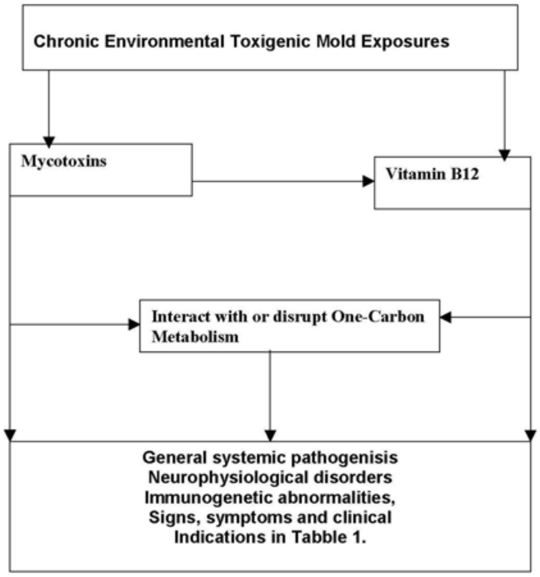

**FIGURE 1:** A proposed outline of the mechanism by which mycotoxins disrupt or interfere with the normal functional effects of vitamin B12 on one-carbon metabolism. It is likely that the interruption of the structure and function of vitamin B12 would in turn interfere with the one-carbon metabolism leading to the neurological manifestations.

## Vitamin B12 in Maintaining a One-Carbon Metabolic Balance

One-carbon metabolism comprises a complex set of reactions that contribute to many central biosynthetic pathways. It is therefore a dynamic metabolic system in which one-carbon units are interconverted between the mitochondrial and cytoplasmic compartments as well as between oxidation states. The importance of this flow has been the subject of extensive biochemical analyses[16,17,18,19,20,21,22,23,24] that showed that glycine, serine, and formate, could each act to supplement all of the different one-carbon pools[11].

The magnitude of the role of vitamin B12 in one-carbon metabolism and the possible effects of mycotoxins on this process can be explained by inferences from Figures 2 and 3 that described the one-carbon metabolism in yeast[11,24]. The process relies on at least one of three one-carbon donor molecules: serine, glycine, or formate. Cells regulate the balance of one-carbon flow between the donors by regulating cytoplasmic serine hydroxymethyltransferase activity in a side reaction occurring in the presence of excess glycine[11]. This control governs the level of 5,10-methylene tetrahydrofolate (5,10-

CH2-H4folate) in the cytoplasm, which has a direct role in signaling transcriptional control of the expression of key genes, particularly those encoding the unique components of the glycine decarboxylase complex (*GCV1*, *GCV2*, and *GCV3*)[11]. It is not readily understood how metabolites flow through the various one-carbon metabolic pathways in the cell and not much is known on mechanisms controlling this flow. However, vitamin B12 achieve this regulatory control probably by altering the balance of one-carbon metabolism between the cytoplasm and the mitochondrion to ensure a constant supply of one-carbon units to the important biosynthetic pathways such as those concerned with purine and pyrimidine biosynthesis. It is most likely that mycotoxins may have inhibitory role in these processes.

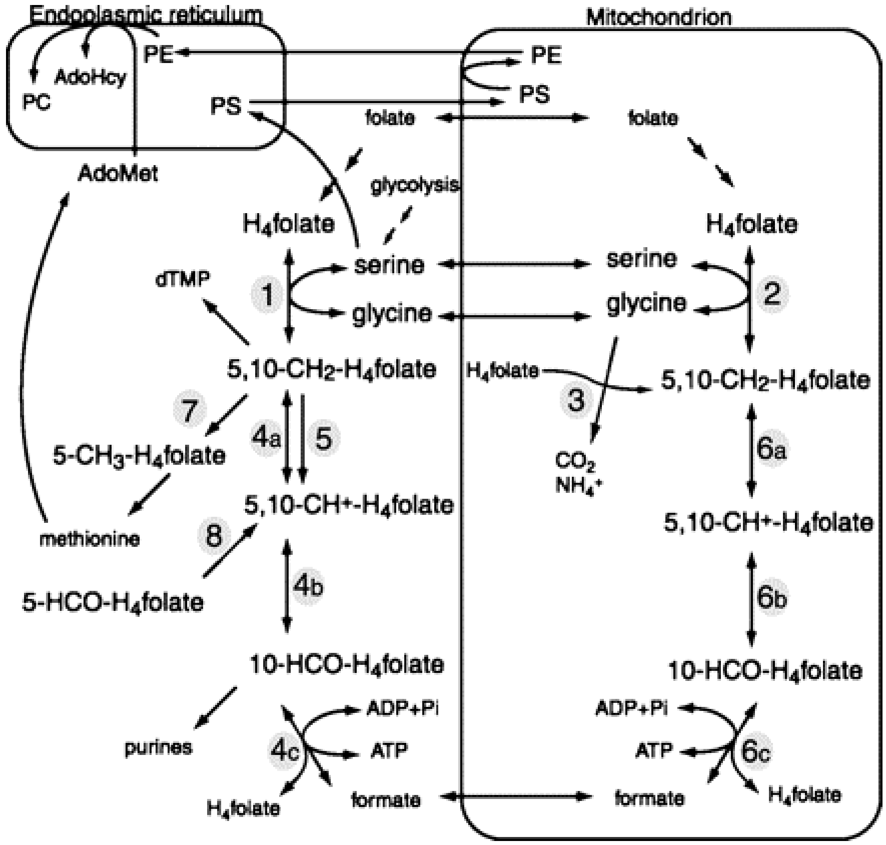

**FIGURE 2.** Intercompartmental flow of one-carbon units in Saccharomyces cerevisiae in the endoplasmic reticulum and the mitochondria. [Note: PC, phosphatidyl choline; AdoHcy, S-adenosylhomocysteine; PE, phosphatidylethanolamine; PS, phosphatidylserine; and AdoMet, S-adenosylmethionine[11].

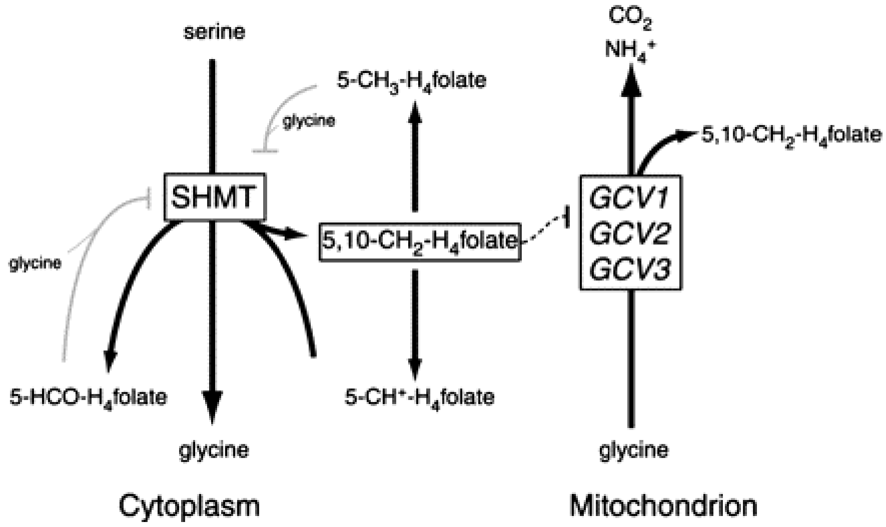

**FIGURE 3.** Model of how cells regulate one-carbon metabolism and gene transcription via the activity of cytoplasmic SHMT. Regulation of SHMT activity by a side reaction dictates cytoplasmic 5,10-CH2-H4folate levels, which mediate transcription of genes involved in generation of one-carbon units from glycine. This control loop has the effect of shifting the balance of one-carbon metabolism toward the mitochondrial reactions. Solid black arrows indicate enzyme-catalyzed reactions; gray lines indicate control of enzyme activity; and the dashed line indicates regulation of gene expression[11]. Permission granted.

## Source of Coenzymes in One-Carbon Metabolism

Vitamin B12 is a coenzyme for two physiologically important functions in humans: 1) the synthesis of methionine, which is a required step in the production of active folate coenzyme, and 2) the conversion of methylmalonic acid to succinic acid. B12 assists in transferring the methyl group onto homocysteine to form methionine, which is necessary for the synthesis of myelin sheaths. Vitamin B12 is required in the methionine synthase reaction in which homocysteine is converted to methionine and methyl tetrahydrofolate (methyl THF) to THF. The 'methyl-folate trap' hypothesis[25] suggests that failure of demethylation of methyl THF with consequent deficiency of folate co-enzymes derived from THF is the crucial lesion caused by vitamin B12 deficiency. Vitamin B12 therefore, serves as a cofactor in the transfer of methyl groups - takes the methyl group from methyl tetrahydrofolate so that THF, the active form of folate, can be used for synthesis of DNA, involved in carbohydrate metabolism. Since vitamin B12 is one of the sources of coenzymes, which participate in one-carbon metabolism, then the interaction with mycotoxins probably affects the coenzymatic action[13]. A more recent theory[25,26] suggests that reduced supply of methionine leads to reduced availability of 'activated formate' and hence of formyl THF and it is the defect that results in failure of folate co-enzyme synthesis. It is in the supply of THF and not of 'active formate' or formyl THF, that vitamin B12 plays a critical role in folate metabolism[25,26]. It is possible that a substantial majority of the cases of high homocysteine in older population can be attributed to vitamin B12 status[12]. Since the metabolism of a substrate is closely linked to that of its cofactor(s), vitamins B12 is most likely involved in homocysteine metabolism, acting as coenzymes[26]. Since homocysteine is an intermediate compound formed during the metabolism of methionine, deficiency of vitamin B12 is one of the major causes of homocysteine elevation in the elderly population[14].

### *Intracellular Recycling of Methionine*

The metabolism and intracellular recycling of methionine requires vitamin B12 as cofactor and methyltetrahydrofolate as co-reactant[27,28,29]. In fact, both vitamin B12 and folic acid are required for the biosynthesis of methionine from homocysteine[30]. The beneficial effects of folate in the above conditions can be explained largely within the context of folate-dependent pathways, such as methionine, purine and pyrimidine biosynthesis[31]. However, the precise detail of folate metabolism is extremely complex and difficult to study because folate-dependent one-carbon metabolism is compartmentalized, involving an enormous number of low-abundance, difficult-to-measure, highly labile folyl coenzymes, and is the subject of genetic variability[31].

## Vitamin B12 in Methylation

Tetrahydrofolate (folic acid derivatives) in conjunction with vitamin B12 involved are in the methylation of homocysteine to methionine. Since this reaction is B12-dependent [27-28] and since mycotoxins interfere with vitamin B12 function (by implication and derivation), there is no doubt that these important biochemical reactions would be affected adversely. Deficiency of vitamin B12 causes serious and often irreversible neurological disorders, such as burning pain or loss of sensation in the extremities, weakness, spasticity and paralysis, confusion, disorientation, and dementia.3 Vitamin B12 is not only required both in the methylation of homocysteine to methionine but also in the synthesis of S-adenosylmethionine. Furthermore, since homocysteine is a sulfhydryl-containing amino acid that is formed by the demethylation of methionine, it is normally catalyzed to cystathionine by cystathionine beta-synthase a pyridoxal phosphate-dependent enzyme and it is also remethylated to methionine by methionine synthase, a vitamin B12 dependent enzyme and by methylenetetrahydrofolate reductase[32]. Vitamin B12 deficiency and genetic defects such as cystathionine beta-synthase or abnormality of methylene-tetrahydrofolate reductase or some vitamin B12 metabolism defects may contribute to increasing plasma homocysteine levels[32]. S-adenosylmethionine is involved in numerous methylation reactions involving proteins, phospholipids, DNA, and neurotransmitter metabolism. Both vitamin B12 and folate deficiency may cause similar neurological and psychiatric disturbances including depression, dementia, and a demyelinating myelopathy. A current theory[33] proposes that a defect in methylation processes is central to the biochemical basis of the neuropsychiatry of these vitamin deficiencies. Vitamin B12 deficiency may specifically affect central monoamine metabolism and aggravate depressive disorders. In addition, the neurotoxic effects of homocysteine may also play a role in the neurological and psychiatric disturbances that are associated with vitamin B12 deficiency[33,34,35].

## Vitamin B12 in the Prevention of Chromosome Breakage

Folic acid plays a critical role in the prevention of chromosome breakage and hypomethylation of DNA. This activity is compromised when Vitamin B12 concentration is low due to reduction in methionine synthase activity and the lowering the concentration of S-adenosyl methionine (SAM). These scenarios, in turn may diminish DNA methylation and cause folate to become unavailable for the conversion[36]. The most plausible explanation for the chromosome-breaking effect of low folate is excessive uracil misincorporation into DNA, a mutagenic lesion that leads to strand breaks in DNA during repair. Both *in vitro* and *in vivo* studies with human cells clearly show that folate deficiency causes expression of chromosomal fragile sites, chromosome breaks, and excessive uracil in DNA, micronucleus formation and DNA hypomethylation[36]. Deficient levels of folic acid and vitamin B12 are associated with elevated chromosome damage rate and high concentrations of homocysteine in the blood. Therefore, elevated homocysteine status, in the absence of vitamin deficiency and low, but not deficient, vitamin B12 status are important risk factors for increased chromosome damage in lymphocytes[37].

## CONCLUSIONS

Vitamin B12 roles in One-carbon metabolism have been discussed. The overall functions of vitamin B12 as a source of coenzymes, in intracellular recycling of methionine, in methionine synthase reaction, in the prevention of chromosome breakage, in methylation and in maintaining a one-carbon metabolic balance have been reviewed. Although, these roles are evidently documented, the effects of mycotoxins on them are yet to be fully realized in the light of the present interests in mycotoxin research. However, the possible adverse interruption of these metabolic roles by mycotoxins have been assessed and evaluated. By implication and derivations from the literatures, it is likely that the interruption of the structure and function of vitamin B12 would in turn interfere with the one-carbon metabolism leading to the neurological manifestations.

## References

1. McCormick, D.B. (1991). Vitamins In: *Fundamentals of clinical chemistry (Third Edition)* Norbert W. Tietz; W.B. Saunders Company.
2. Kaplan, L.A. and Pesce, A.J. (Eds.) (1989). *Clinical Chemistry, Theory, analysis, and correlation (2nd ed.).* The C.V. Mosby Company.
3. Fairbank, V.F. and Klee, G.G. (1991). Biochemical aspects of hematology. In: Norbert W. Tietz (ed.), *Fundamentals of Clinical Chemistry, (Third Edition).* WB Saunders, London.
4. Riley, R.T., Enongene, E., Voss, K.A., Norred, W.P., Meredith, F.I., Sharma, R.P., Spitsbergen, J., Williams, D.E., Carlson, D.B., and Merrill, A.H. Jr. (2001). Sphingolipid perturbations as mechanisms for fumonisin carcinogenesis. *Environ. Health Perspect*. **109 (Suppl 2)**. 301-308.
5. Enongene, E.N., Sharma, R.P., Bhandari, N., Voss, K.A., and Riley, R.T. (2000). Disruption of sphingolipid metabolism in small intestines, liver and kidney of mice dosed subcutaneously with fumonisin B(1). *Food Chem. Toxicol*. **38(9).** 793-799.
6. Crews, H., Alink, G., Andersen, R., Braesco, V., Holst, B., Maiani, G., Ovesen, L., Scotter, M., Solfrizzo, M., van den Berg, R., Verhagen, H., and Williamson, G. (2001). A critical assessment of some biomarker approaches linked with dietary intake. *Br. J. Nutr*. **86 (Suppl 1).** S5-35.
7. Samaranayake, L.P. (1986). Nutritional factors and oral candidosis. J. Oral Pathol. **15(2).** 61-65.
8. Hitzig, W.H. (1983). Protean appearances of immunodeficiencies: syndromes and inborn errors involving other systems which express associated primary immunodeficiency. *Birth Defects Orig. Artic. Ser.* **19(3).** 307-312
9. Samaranayake, L.P. (1981). MacFarlane TW. A retrospective study of patients with recurrent chronic atrophic candidosis. *Oral Surg. Oral Med. Oral Pathol.* **52(2).** 150-153.
10. Challacombe, S.J. (1986). Haematological abnormalities in oral lichen planus, candidiasis, leukoplakia and non-specific stomatitis. *Int. J. Oral Maxillofac. Surg.*, **15(1).** 72-80.
11. Piper, M.D., Hong, S., Ball, G.E., and Dawes, I.W. (2000). Regulation of the Balance of One-carbon Metabolism in Saccharomyces cerevisiae. *J. Biol. Chem.* **275 (40).** 30987-30995.
12. Selhub, J., Jacques, P.F., Wilson, P.W., Rush, D., and Rosenberg, I.H. (1993). Vitamin status and intake as primary determinants of homocysteinemia in an elderly population. *J.A.M.A.* **270(22).** 2693-2698.
13. Selhub, J. (2002). Folate, vitamin B12 and vitamin B6 and one carbon metabolism. *J. Nutr. Health Aging.* **6(1).** 39-42.
14. Moustapha, A. and Robinson, K. (1998). High plasma homocysteine: a risk factor for vascular disease in the elderly. *Coron. Artery Dis.* **9(11).** 725-30.
15. Wainfan, E. and Poirier, L.A. (1992). Methyl groups in carcinogenesis: effects on DNA methylation and gene expression. *Cancer Res.* **52(7 Suppl)**. 2071s-2077s.
16. McKenzie, K.Q. and Jones, E.W. (1977). Mutants of formyltetrahydrofolate interconversion pathway of Saccharomyces cerevisiae. *Genetics.* **86(1).** 85-102.
17. Ogur, M., Liu, T.N., Cheung, I., Paulavicius, I., Wales, W., Mehnert, D., and Blaise, D. (1977). "Active" one-carbon generation in Saccharomyces cerevisiae. *J. Bacteriol.* **129(2).** 926-933.
18. Schirch, L. (1984). In: *Folates and Pterins*. Blakley, R. L., and Benkovic, S. J. (eds.), John Wiley & Sons, Inc., New York. ***Vol. 1.*** 399-431.
19. Barlowe, C.K. andAppling, D.R. (1988). In vitro evidence for the involvement of mitochondrial folate metabolism in the supply of cytoplasmic one-carbon units. *Biofactors.* **1(2).** 171-176.
20. Pasternack, L.B. and Laude, D.A. Jr. (1994). Appling DR.13C NMR analysis of intercompartmental flow of one-carbon units into choline and purines in Saccharomyces cerevisiae. *Biochemistry* **33(1).** 74-82.

21. Pasternack, L.B., Laude, D.A. Jr., and Appling, D.R. (1994). Whole-cell detection by 13C NMR of metabolic flux through the C1-tetrahydrofolate synthase/serine hydroxymethyltransferase enzyme system and effect of antifolate exposure in Saccharomyces cerevisiae. *Biochemistry.* **33(23).** 7166-7173.
22. Pasternack, L.B., Littlepage, L.E., Laude, D.A. Jr., and Appling, D.R. (1996). 13C NMR analysis of the use of alternative donors to the tetrahydrofolate-dependent one-carbon pools in Saccharomyces cerevisiae. *Arch. Biochem. Biophys.* **326(1).** 158-165.
23. Pasternack, L. B., Laude, D. A., and Appling, D. R. (1992) 13C NMR detection of folate-mediated serine and glycine synthesis in vivo in Saccharomyces cerevisiae. *Biochemistry.* **31(37).** 8713-8719.
24. West, M.G., Horne, D.W., and Appling, D.R. (1996). Metabolic role of cytoplasmic isozymes of 5,10-methylenetetrahydrofolate dehydrogenase in Saccharomyces cerevisiae. *Biochemistry.* **35(9).** 3122-3132.
25. Hoffbrand, A.V. and Jackson, B.F. (1993). Correction of the DNA synthesis defect in vitamin B12 deficiency by tetrahydrofolate: evidence in favour of the methyl-folate trap hypothesis as the cause of megaloblastic anaemia in vitamin B12 deficiency. *Br. J. Haematol.* **83(4).** 643-647.
26. Pietrzik, K., Bronstrup, A. (1998). Vitamins B12, B6 and folate as determinants of homocysteine concentration in the healthy population. Eur. J. Pediatr. **157 (Suppl. 2).** S135-8.
27. Gregory, J.F. 3rd., Cuskelly, G.J., Shane, B., Toth. J.P., Baumgartner, T.G., and Stacpoole, P.W. (2000). Primed, constant infusion with [2H3]serine allows in vivo kinetic measurement of serine turnover, homocysteine remethylation, and transsulfuration processes in human one-carbon metabolism. *Am. J. Clin. Nutr.* **72(6).** 1535-1541.
28. Gregory, J.F. 3rd., Swendseid, M.E., and Jacob, R.A. (2000). Urinary excretion of folate catabolites responds to changes in folate intake more slowly than plasma folate and homocysteine concentrations and lymphocyte DNA methylation in postmenopausal women. *J. Nutr.* **130(12).** 2949-2952.
29. Maree, K.A., van der Westhuyzen, J., and Metz, J. (1990). Interrelationship between serum concentrations of methionine, vitamin B12 and folate. *Int. J. Vitam. Nutr. Res.* **60(2).** 136-141.
30. Lu, N.C., Hieb, W.F., and Stokstad, E.L. (1976). Effect of vitamin B12 and folate on biosynthesis of methionine from homocysteine in the nematode Caenorhabditis briggsae. *Proc. Soc. Exp. Biol. Med.* **151(4).** 701-706.
31. Lucock, M. and Daskalakis, I. (2000). New perspectives on folate status: a differential role for the vitamin in cardiovascular disease, birth defects and other conditions. *Br. J. Biomed Sci.* **57(3).** 254-260.
32. Nicolas, J.P. and Chango, A. (1997). Deregulation of homocysteine metabolism and consequences for the vascular system. *Bull. Acad. Natl. Med.* **181(2).** 313-329; discussion 330-331.
33. Bottiglieri, T. (1996). Folate, vitamin B12, and neuropsychiatric disorders. *Nutr. Rev.* **54(12).** 382-390
34. Verhoef, P., Stampfer, M.J., Buring, J.E., Gaziano, J.M., Allen, R.H., Stabler, S.P., Reynolds, R.D., Kok, F.J., Hennekens, C.H., and Willett, W.C. (1996). Homocysteine metabolism and risk of myocardial infarction: relation with vitamins B6, B12, and folate. *Am. J. Epidemiol.* **143(9).** 845-859.
35. Martinez, M., Cuskelly, G.J., Williamson, J., Toth, J.P., and Gregory, J.F. 3rd. (2000). Vitamin B-6 deficiency in rats reduces hepatic serine hydroxymethyltransferase and cystathionine beta-synthase activities and rates of in vivo protein turnover, homocysteine remethylation and transsulfuration. *J. Nutr.* **130(5).** 1115-1123.
36. Fenech, M. (2001). The role of folic acid and Vitamin B12 in genomic stability of human cells. *Mutat. Res.* **475(1-2).** 57-67.
37. Fenech, M.F., Dreosti, I.E., and Rinaldi, J.R. (1997). Folate, vitamin B12, homocysteine status and chromosome damage rate in lymphocytes of older men. *Carcinogenesis* **18(7).** 1329-1336.
38. Anyanwu, E.C., Morad, M., and Campbell, A.W. (2004) Metabolism of mycotoxins, intracellular functions of vitamin B12, and neurological manifestations in patients with chronic toxigenic mold exposures. A review. *TheScientificWorldJOURNAL* **4**, 736-745.
